# Endophthalmitis in Retinopathy of Prematurity after Intravitreal Aflibercept Injection

**DOI:** 10.1155/2020/8861435

**Published:** 2020-12-21

**Authors:** Elcin Suren, Ersan Cetinkaya, Mustafa Kalayci, Sariye Elif Özyazıcı Özkan, Mehmet Fatih Kucuk, Muhammet Kazim Erol

**Affiliations:** ^1^University of Health Sciences, Antalya Training and Research Hospital, Department of Ophthalmology, Antalya, Turkey; ^2^University of Health Sciences, Antalya Training and Research Hospital, Department of Pediatrics, Antalya, Turkey

## Abstract

We report the case of a male infant who had an intravitreal anti-VEGF (aflibercept) injection for the treatment of retinopathy of prematurity at 35-week postmenstrual age. Four days following the injection, retinal imaging demonstrated a yellowish gray blurred mass that extended into the vitreous in the right eye, and the vitreous body was blurred. After two days, despite starting endophthalmitis treatment, there was still no improvement in the retinal lesion. Due to the worsening of the clinical signs, we decided to perform 25-gauge lens-sparing pars plana vitrectomy.

## 1. Introduction

Retinopathy of prematurity (ROP) is caused by an abnormality in the development of blood vessels in premature babies. This leads to potential vision loss. It is an important cause of childhood blindness in both developed and developing countries [1]. Peripheral retinal ablation with confluent pattern laser therapy is the traditional treatment for ROP; however, this therapy is destructive, invasive, and not entirely effective against blindness in premature babies [2]. Today, antivascular endothelial growth factor (VEGF) injections stand out as an alternative and up-to-date therapy with good results [3].

Infectious endophthalmitis is the most destructive and scary complication of intravitreal anti-VEGF injections [4]. To our knowledge, the literature contains only two case reports on infectious endophthalmitis after anti-VEGF injections in ROP [4, 5]. Our purpose in this case report is to report the first case of endophthalmitis that developed after aflibercept injection in ROP and to discuss the management of this complication.

## 2. Case Report

A male infant with a gestational age of 27 weeks and a birth weight of 1,200 g was admitted to the ROP unit of ATR Hospital. A routine retinal screening at 31-week postmenstrual age (PMA) indicated a demarcation line in the posterior retina. Two weeks later, the ridge became marked, and stage 1-2 ROP without plus disease was defined. At 35-week PMA, retinal disease progressed with the retinal image demonstrating stage 2-3 ROP in zone 2 with plus disease (tortuous arteries and dilated veins) in the posterior retina. At this visit, we decided to treat the patient. The parents of the patient were written and verbally informed about the side effects of the treatment options, and it was decided to apply an intravitreal anti-VEGF (aflibercept) injection for the treatment of ROP.

**Figure 1:**
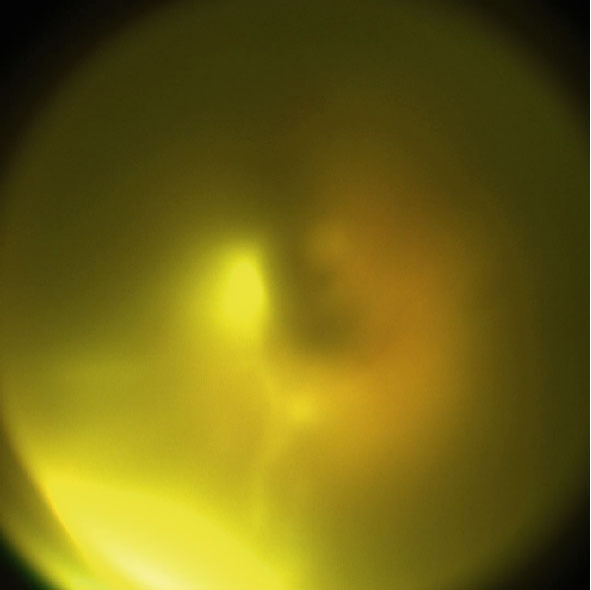
Yellowish-gray blurred mass that extended into the vitreous.

**Figure 2:**
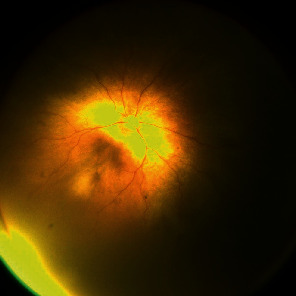
Favorable anatomic result was achieved four weeks after the pars plana vitrectomy.

Written informed consent was obtained from the parents before the intravitreal injection. The injection was administered in an operating room. A topical anesthetic, 5 mg proparacaine HCl (Alcaine), was applied to both eyes, and a 5% povidone-iodine solution was applied for surgical asepsis. Then, aflibercept was injected 1.5 mm posterior to the limbus in each eye. As followed in our routine procedure after surgery, a topical antibiotic (moxifloxacin [0.5%] ophthalmic solution) and steroid (dexamethasone ophthalmic solution) combination drop was recommended eight times daily. No systemic antibiotic was applied. Four days following the injection, plus disease and ROP stage were regressed in the left eye but retinal imaging demonstrated a yellowish-gray blurred mass that extended into the vitreous in the right eye, and the vitreous body was blurred (Figure 1). In the right eye, no conjunctival congestion or hypopyon was observed. We diagnosed endophthalmitis based on the characteristics of the retinal lesion and immediately administered empirical therapy through an intravitreal injection of vancomycin (0.5 mg) and ceftazidime (1 mg) to the right eye (half of the adult dosage) after a vitreous tap. Intravenous meropenem (40 mg/kg∗3), vancomycin (15 mg/kg∗3), and amikacin (15 mg single dose) were also started in the neonatal intensive care unit after requesting blood culture analysis. After two days, despite starting endophthalmitis treatment, there was still no improvement in the retinal lesion. The vitreous sample and blood culture were negative for microbiological analysis. Corneal edema started. Due to the worsening of the clinical signs, we decided to perform 25-gauge lens-sparing pars plana vitrectomy (PPV) in the right eye after obtaining informed consent from the parents. The vitreous membranes were removed. Three days after the surgery, retinal imaging demonstrated a decrease in the size of the retinal lesion, the retina was partially clearly seen, and neither ROP nor plus disease was noted. Topical drops and intravenous antibiotics were continued for 10 days. A favorable and satisfactory anatomic result was achieved four weeks after the surgery (Figure 2).

## 3. Discussion

Anti-VEGF treatment has numerous advantages for the treatment of ROP. However, intravitreal injections may cause noninfectious or infectious intraocular inflammatory reactions [6]. In major studies, no endophthalmitis associated with intravitreal injection has been reported in ROP. To our knowledge, there are two reported cases of endophthalmitis in ROP. Wang and Xiang [4] defined bacterial endophthalmitis in ROP after an intravitreal bevacizumab injection, and Chandra et al. [5] presented a case after an intravitreal ranibizumab injection. We reported the first case of postaflibercept injection endophthalmitis in ROP. Endophthalmitis in infants may involve many challenges, such as the lack of prominent clinical features (e.g., pain, chemosis, edema, and hypopyon), delayed presentation, and difficulty in differentiating between sterile and infectious inflammation. In adults, there are numerous studies showing sterile inflammation after an intravitreal aflibercept injection with favorable outcomes [7]. In contrast, even a suspect of endophthalmitis in ROP should be managed by hospitalization. In our case, only the right eye was inflamed, and despite the absence of other features in endophthalmitis, the case did not appear to have sterile inflammation; thus, it was evaluated as endophthalmitis induced by the intravitreal aflibercept injection. As endophthalmitis may seriously damage vision, it has been suggested that broad-spectrum intravitreal antibiotics, ceftazidime plus vancomycin, should be applied to treat this condition before the differentiation of infective organisms. In addition, intravenous therapy with a wide antimicrobial coverage should be started. Amikacin, meropenem, and tazobactam–piperacillin seem to be ideal empirical systemic antibiotics with higher intravitreal penetration in infants. Studies have shown that an early intravitreal injection of broad-spectrum antibiotics may be an effective and important treatment for premature infants with bacterial endophthalmitis [7, 8]. However, endophthalmitis in ROP necessitates early surgery if the patient does not respond to medical therapy. In our present case, the infection could not be controlled by medical therapy. In cases involving postoperative bacterial endophthalmitis, only 69% of the cultures show positive bacterial growth [9]. Therefore, despite the negative results obtained from the vitreous and blood culture, we accepted the case as infectious. We evaluated no clinical improvement and performed PPV. The role of PPV in the management of endophthalmitis in the pediatric population has been emphasized in previous studies [9]. PPV may be reserved because of higher risk of ocular complications and anesthesia-related mortality and morbidity of endophthalmitis in infants but early PPV and intravitreal vancomycin plus ceftazidime injections have been shown to improve functional and anatomic success in pediatric eyes with exogenous endophthalmitis [10].

The current case also indicates that early PPV may be effective for the management of endophthalmitis in ROP after an intravitreal injection. In conclusion, an intravitreal injection of broad-spectrum antibiotics is administered in medical therapy for endophthalmitis in ROP as the first-line treatment but infants who are unable to complain of a loss of visual acuity may need to be treated by early PPV and intravitreal vancomycin plus ceftazidime injections to improve positive outcomes.

## Data Availability

Data is available on request.
